# Analysis of transcriptome characteristics of UTI therapy for cerebral injury after CA/ROSC based on RNA-seq technique

**DOI:** 10.22038/IJBMS.2022.61990.13722

**Published:** 2022-06

**Authors:** Xiaojie Bai, Mingya Yang, Tiantian Zhu, Jun Xu, Wei Wang, Yuanyuan He, Yu Liu, Xingxing Li, Miao He, Tao Meng, Zhenzhen Wang, Hong Zhang, Lixin Zhu

**Affiliations:** 1 Department of Emergency, the First Affiliated Hospital of Anhui Medical University, Hefei 230022, PR China; 2 Department of Hematology, the First Affiliated Hospital of Anhui Medical University, Hefei 230022, PR China; 3 Department of General Surgery, the First Affiliated Hospital of Anhui Medical University, Hefei 230022, PR China

**Keywords:** Brain injuries, Cardiopulmonary – resuscitation, Heart arrest, RNA-Seq, Urinastatin

## Abstract

**Objective(s):**

To study the effects and mechanisms of ulinastatin (UTI) on brain injury caused by cardiac arrest/return of spontaneous circulation (CA/ROSC).

**Materials and Methods:**

In this study, modeling of CA/ROSC was set up in 56 Sprague Dawley (SD) rats, which were randomly divided into the model group, UTI (100000U/kg) treatment group, and control group. Each group then was divided into two subgroups: 24 hr and 72 hr. The survival rates between different groups was observed during two weeks. AimPlex multiplex immunoassays technology was performed to detect the expression of inflammatory cytokines in serum, such as IL-6 and TNF-α. RNA-sequencing (RNA-seq) transcriptome, Gene Ontology (GO), and Kyoto. Encyclopedia of Genes and Genomes (KEGG) enrichment analysis were used to investigate the possible mechanism of UTI. Western blot and immunohistochemistry were performed to detect the expression of C-C motif chemokine ligand 2 (CCl2) and plasminogen (plg) protein expression.

**Results:**

The survival rate of the UTI group was significantly higher than the model group during two weeks. And UTI can significantly reduce the content of IL-6 and TNF-α in serum. GO and KEGG pathway enrichment analysis revealed that differentially expressed genes mainly belonged to the IL-17 signaling pathway and neuroactive ligand-receptor interaction signaling pathway. Besides, UTI can down-regulate the expression of the CCl2 inflammatory gene and up-regulate the expression of plg in the brain tissue of CA/ROSC rats.

**Conclusion:**

UTI has neuroprotective effects on brain injury after CA/ROSC. And the key mechanisms belong to the regulation of immune-inflammatory response as well as the signaling molecules and interaction.

## Introduction

CA is a critical disease with a poor prognosis, brain damage after CA is one of the main pathophysiological changes of the post-CA syndrome (1). Although the success rate of ROSC has been increased based on the improvement of the cardiopulmonary resuscitation (CPR) technique, the prognosis is far from satisfactory. Reducing brain damage after CA has become a key issue to improve the prognosis (2, 3). In previous research, researchers had proposed many different treatments for acute ischemic brain injury, which included drug therapy, gene therapy, hyperbaric oxygen therapy, music therapy, mild hypothermia therapy, and so on (4-10). However, their effects were limited. Currently, there are no effective drugs or technologies for brain injury after CA/ROSC, which not only afflicts medical workers but also increases the burden on society and families. Thus, it is important to find new interventions and further study their mechanisms. 

UTI is a protease inhibitor modulating inflammatory, oxidative stress response, neuronal apoptosis, autophagy, and blood-brain barrier permeability (11, 12). It has been widely used in therapy for sepsis, severe acute pancreatitis, and multiple organ dysfunction (13-15). Some studies have also confirmed that UTI can improve various kinds of brain injuries (16, 17), but the mechanism is unclear. As a newly developed high-throughput transcriptome sequencing technology in recent years, RNA-seq has been widely applied in the identification of DEGs and transcripts (18). Therefore, to further elaborate on the major mechanism of brain injury after CA/ROSC, RNA-seq technology was practiced in this study. In addition, GO and KEGG enrichment analysis, AimPlex multiplex immunoassays technology, western blot (WB), and immunohistochemistry (IHC) were also used to investigate the effect of UTI on brain injury after CA/ROSC and the possible mechanism.

## Materials and Methods

***Experimental design***

**Male SD rats (300**±15 g) were purchased from Jinan Pengyue Experimental Animal Breeding Co. Ltd (registration number: 370181000009090). All animals were housed in 22–25 °C, humidity‐controlled (50% relative humidity), and 12 hr light/dark cycle conditions with food and water available. The experimental protocol was approved by the Animal Committee of Anhui Medical University and conducted in accordance with the requirements of the Guide for the Care and Use of Laboratory Animals.

Fifty-six adult male SD rats were randomly divided into three groups: control group (n=12), model group (n=22), and UTI treatment group (n=22). Then model group and UTI treatment group were divided into two subgroups respectively: 1d and 3d. After acclimatization for 3 days, rats in the model and UTI group underwent asphyxia-CA for 6 min. In the UTI group, UTI (100000 U/kg) was injected intravenously within 2 min after successful CPR. There was no treatment in the control group. All animals then were sacrificed at 1d and 3d, respectively. 

***Modeling of CA/ROSC***

**Figure 1:**
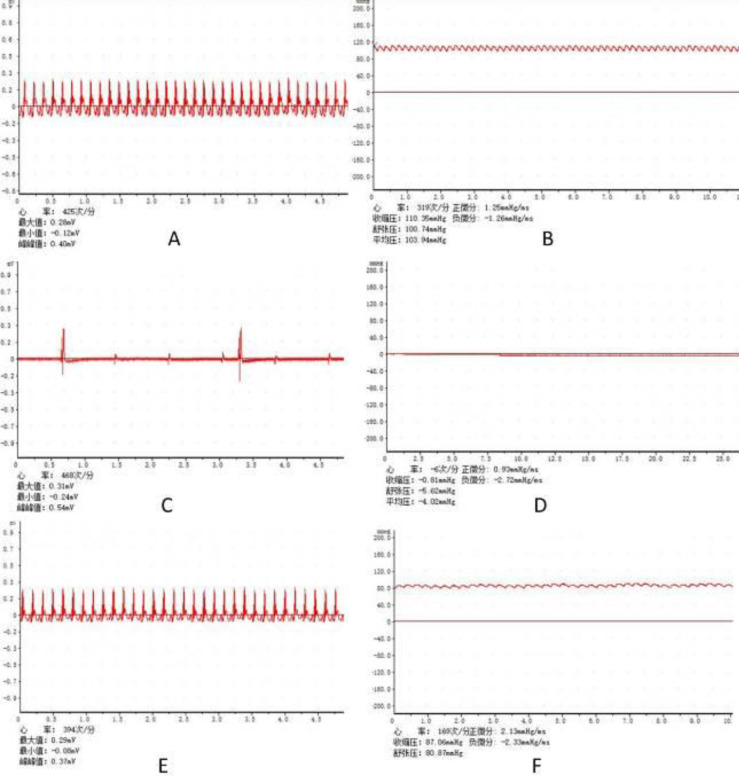
Differences of MAP and ECG in the process of a rat model of CA/ROSC. (A) Normal ECG before CA; (B) Normal MAP before CA; (C) ECG when CA occurs; (D) MAP when CA occurs; (E) ECG after ROSC; (F) MAP after ROSC

MAP: mean arterial pressure; ECG: electrocardiogram; CA: cardiac arrest; ROSC: return of spontaneous circulation

The subjects were fasted overnight and anesthetized with 10% chloral hydrate (0.35 ml/100 g), intraperitoneally. The rats were fixed and connected to an electrocardiogram (ECG, Figure 1A). Then endotracheal intubation was performed with a breathing catheter. Controlled intermittent positive pressure ventilation (HX-200, Chengdu Taimeng Technology Co. Ltd) with tidal volumes of 1.0 ml /100 g and inhalation /expiratory ratio (1/2) at a rate of 70 breaths/min was used. After separating the femoral artery and femoral vein for arteriovenous catheterization, the femoral vein was used for drug administration, while the femoral artery was for continuous monitoring of mean arterial pressure (MAP, Figure 1B). The T-branch pipe was connected to the remaining arterial needle, while one end was connected to the physiological monitoring recorder (BL-420F, Chengdu Taimeng Technology Co. Ltd). 

After monitoring the ECG and MAP for 15 min, vecuronium (0.1 ml/100 g) was injected through the femoral vein, mechanical ventilation was stopped and the respiratory catheter was blocked about 30 sec later. The criteria for CA: ①The ECG wave indicated ventricular fibrillation, pulseless electrical, or asystole (Figure 1C); ②The MAP decreased below 20 mmHg (Figure 1D). CPR was initiated at 6 min after CA and lasted for a maximum of 10 min. Chest compressions were carried out at a rate of 200–300 compressions per minute with the depth of compression to 1/3 of the anteroposterior chest diameter. Epinephrine (0.2 ml/100 g) was immediately administrated at the beginning of CPR and repeated at 2 min if necessary. The criteria for ROSC: ①The ECG returned to normal (Figure 1E); ②The MAP was more than 60 mmHg for at least 10 min (Figure 1F).

***Survival rates***

**The survival rates of ten samples in the model group and the UTI group were observed and recorded within 2 weeks.**

***Extraction of brain tissue***

Rats in each group were sacrificed at the corresponding points and the samples were collected. Brain tissue was perfused with normal saline before extraction. Fresh brain tissue was frozen and stored in a refrigerator at -80 °C after being separated into the left and right hemispheres. The left hemisphere was sent for RNA-seq while the right one was for WB. Part of the brain tissue was used to make paraffin specimens. 

***RNA-Seq analysis***

Total RNA was isolated from each brain sample with the standard Trizol protocol (Invitrogen, Carlsbad, CA, USA). RNA quality was examined by gel electrophoresis and a Nanodrop spectrophotometer (Thermo, Waltham, MA, USA). Strand-specific libraries were constructed using the TruSeq RNA sample preparation kit (Illumina, San Diego, CA, USA), and sequencing was carried out using the Illumina novaseq-6000 instrument by the commercial service of Genergy Biotechnology Co. Ltd. (Shanghai, China). The raw data was handled by Perl and data quality was checked by FastQC v0.11.2. The thresholds for determining differentially expressed genes (DEGs) were *P*<0.05 and absolute fold change≥1. Then DEGs were chosen for function and signaling pathway enrichment analysis using GO and KEGG databases. The significantly enriched pathways were determined when *P*<0.05 and at least two affiliated genes were included. IHC and WB were used to validate the expression of candidate genes in UTI therapy and model groups. 

***Aimplex multiplex immunoassays technology***

Serum levels of interleukin-6 (IL-6) and tumor necrosis factor-alpha (TNF-α) were detected with flow cytometry (Rat Custom 6-plex kit, Beijing kuangbo biotechnology co., LTD). Briefly, each bead in a population was conjugated with capture antibodies specific to one cytokine. This antibody traps the protein of interest from the sample. The amount of the analyte captured is detected via a biotinylated antibody against a secondary epitope of the protein, followed by streptavidin-R-phycoerythrin (PE) treatment. Concentrations of cytokines in the samples were determined by comparing the fluorescent signals of samples against a standard curve generated from serial dilutions of known concentrations of the analysis. The assay protocol consists of a 60-min incubation step to allow antigen capture by antibody conjugated bead, a 30-min incubation step for biotinylated-antibody detection of analysis and a 20-min streptavidin-PE incubation step. The fluorescence intensities of the beads were measured using a NovoCyte D1040 flow cytometer (Aisen Biological Co., Ltd). Each sample was tested in duplicate and the mean of the three samples was used for analysis. The data was processed using FCAP Array 3.0 software and each cytokine concentration was expressed in pg/ml.

***WB analysis***

Total protein was extracted from the collected brain samples using RIPA lysate, and protein concentration was determined using a BCA protein assay kit (Beyotime Biotech Inc). Total protein was separated by 10% sodium dodecyl sulfate-polyacrylamide gel electrophoresis and transferred to a polyvinylidene fluoride membrane. Then membrane blocking was performed with Tris-buffered saline with Tween20 encompassing 5% skim milk powder at room temperature. Overnight membrane incubation was implemented at 4 °C with primary antibodies against plg (abcam, 1:4000, ab154560), CCl-2 (Abmart, 1:4000, MB0556), and bactin (Abmart, 1:4000, ). Secondary goat anti-rabbit (Abmart, 1:8000, M21002) or goat anti-mouse (Abmart, 1:8000, M21001) antibodies were added and the membrane was incubated at ambient temperature for 1 hr. Electrogenerated chemiluminescence was applied to develop the protein bands, followed by imaging with a gel imager (Tanon-5200Multi, China). The expression levels of target proteins were evaluated as the ratios of the detected gray values of the target proteins to that of bactin (ImageJ).

***IHC***

After dewaxing, brain sections were boiled (in an 850 W microwave oven) for 24 min in citrate buffer (3.23 g/l, pH 6.0, Beijing Zhongshan Golden Bridge Biotechnology Co, Ltd., Beijing, China). Endogenous peroxidase inhibitor was incubated at 37 °C (Beijing Zhongshan Golden Bridge Biotechnology Co, Ltd., Beijing, China) for 15 min. Sections were incubated with the mouse monoclonal antibody against CCl-2 (1:200; Abmart, China) and rabbit polyclonal antibody against plg (1:200; Abcam, China) overnight at 4 °C. Subsequently, sections were then incubated in appropriate secondary antibodies (Beijing Zhongshan Golden Bridge Biotechnology Co, Ltd., Beijing, China) for 30 min at room temperature. Washing with PBS three times for 5 min each before proceeding to the next step. 3,3’-diaminobenzidine (Beijing Zhongshan Golden Bridge Biotechnology Co, Ltd., Beijing, China) was used as a chromogen to make the color reaction. Finally, sections were counterstained with hematoxylin. Due to the CA1 area of the hippocampus being usually the most vulnerable structure in ischemia-hypoxia (19), the cerebral cortex and CA1 were mainly observed. 

***Statistical analysis***

The data generated by RNA-seq were mapped onto the reference genome and the quality of the transcriptome library was evaluated according to those results. The different information between different groups was screened based on the expression quantitative results, and significant DEGs were defined as those with P<0.05 and |log2foldchange| ≥1. The other data were all processed by SPSS 23.0 using t-test or one-way analysis of variance and measurement data were expressed as mean ± standard deviation (x±s). The difference is statistically significant when P<0.05.

## Results

***Differences in survival rates between groups***

**Figure 2:**
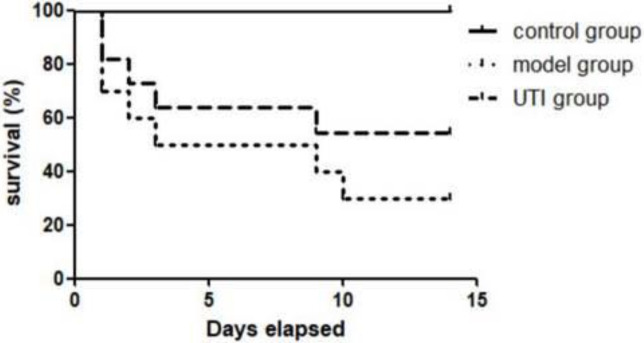
Survival rates of rats in different groups within two weeks

The survival rate of rats in the control group was 100% within 2 weeks. The survival rates in the model group were 70% (7/10), 50%(5/10), 50%(5/10), 30%(3/10), 30%(3/10), and 30%(3/10) on 1d, 3d, 6d, 10d, and 14d after modeling, respectively. The survival rates in the UTI group on 1d, 3d, 6d, 10d, and 14d were 80% (8/10), 60%(6/10), 60%(6/10), and 50%(5/10), 50%(5/10), respectively (Figure 2).

***DEGs between UTI and model groups***

**Figure 3:**
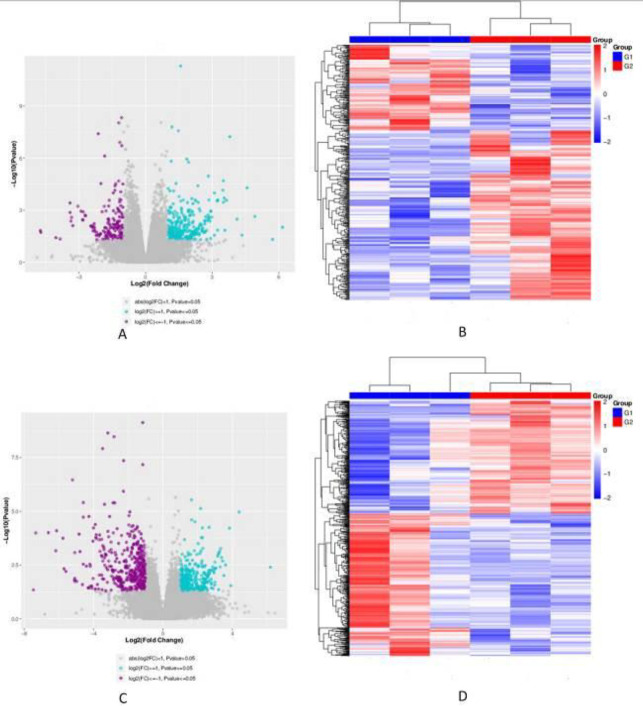
DEGs between UTI and model groups. (A) Volcano plot of UTI 24 hr group vs model 24 hr group; (B) Heat map of DEGs between model 24 hr group and UTI 24 hr group. (C) Volcano plot of UTI 72 hr group vs model 72 hr group. (D) Heat map of DEGs between model 72 hr group and UTI 72 hr group

DEGs were analyzed based on the sequencing results between UTI and model groups. There were 296 up-regulated genes (blue dots) and 150 down-regulated genes (purple dots) between UTI 24 hr and model 24 hr groups (Figure 3A). And between UTI 72 hr and model 72 hr groups, 340 up-regulated genes, and 427 down-regulated genes changed significantly (Figure 3C).

In the heat map, the color change from blue to red indicates that the gene expression changes are from low to high, and the visual contrast is obvious and regular after UTI intervention on 24 hr and 72 hr, respectively (Figures 3B, 3D).

***GO enrichment analysis***

*UTI 24 hr and model 24 hr groups*

**Figure 4:**
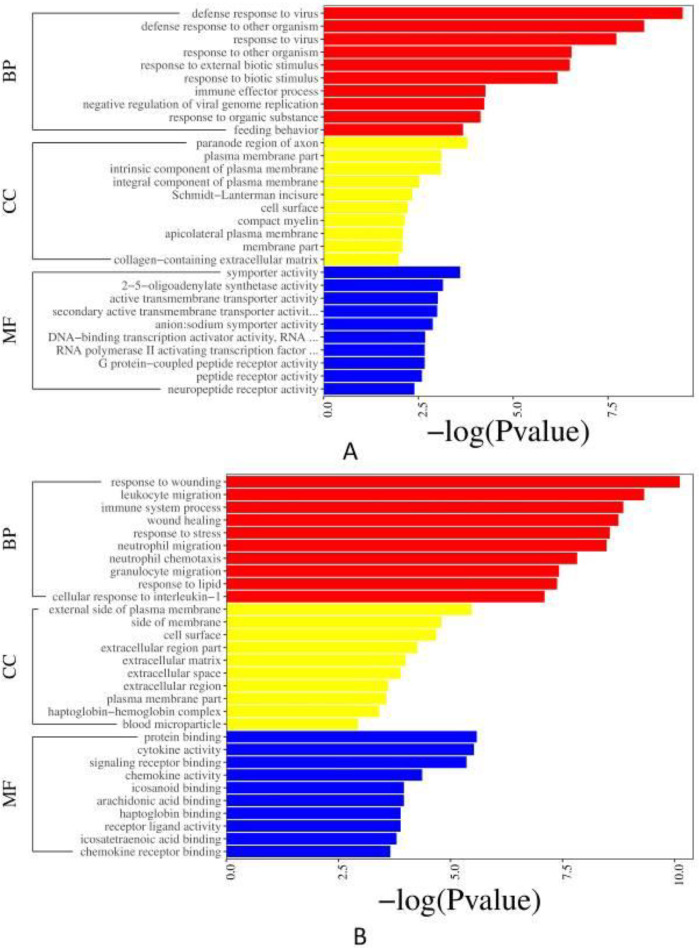
GO enrichment. (A) Bar plot of down-regulated DEGs between UTI 24 hr and model 24 hr groups; (B) Bar plot of up-regulated DEGs between UTI 24 hr and model 24 hr groups

GO: Gene Ontology; UTI: ulinastatin; DEGs: differential expression genes; BP: biological process; CC: cellular component; MF: molecular function

The GO term of down-regulated DEGs (*P*<0.05, Figure 4A) is mainly enriched in the BP, including defense response to viruses or other organisms. And in the classification of CC and MF, the paranode region of axon and symporter activity account for the most, respectively. The GO term of up-regulated DEGs (*P*<0.05, Figure 4B) is also mainly enriched in the BP, including response to wounding, leukocyte migration, immune system process, etc. Regarding the classification of CC, there were sequences associated with the external side of the plasma membrane, side of the membrane, and cell surface, which represented the most abundant categories. The sequences associated with protein binding, cytokine activity, and signaling receptor binding were dominant in the MF category. 

*UTI 72 hr and model 72 hr groups*

**Figure 5:**
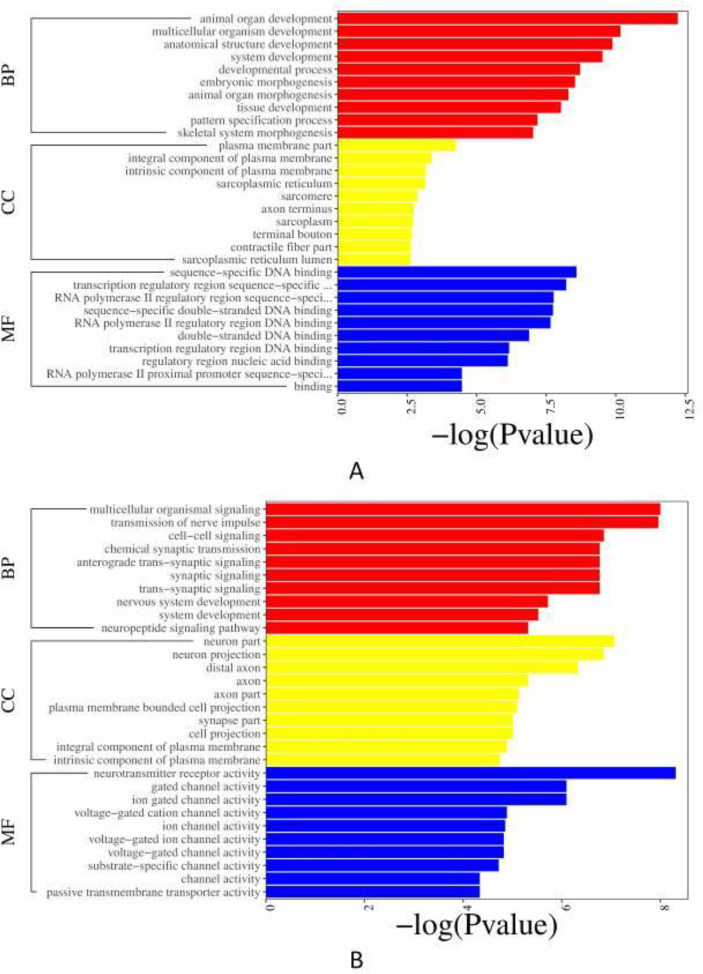
GO enrichment. (A) Bar plot of down-regulated DEGs between UTI 72 hr and model 72 hr groups; (B) Bar plot of up-regulated DEGs between UTI 72 hr and model 72 hr groups

GO: Gene Ontology; UTI: ulinastatin; DEGs: differential expression genes. BP: biological process; CC: cellular component; MF: molecular function

The GO term of down-regulated DEGs (*P*<0.05, Figure 5A) mainly enriched in the BP, terms related to the development of animal organs, anatomical structure, and embryonic morphogenesis in the BP were predominant, followed by the MF mainly containing sequence-specific DNA binding, transcription regulatory region sequence-specific DNA binding, etc. In the classification of CC, the plasma membrane part was primarily enriched. The GO enrichment results of the up-regulated DEGs are as follows (*P*<0.05, Figure 5B). They influenced the BP which mainly contains multicellular organismal signaling, transmission of nerve impulse, cell-cell signaling, etc. The neuron part, neuron projection, and distal axon were the major representation of CC. More enriched GO terms in the MF mainly included neurotransmitter receptor activity, gated channel activity, etc.

***KEGG pathway analysis***

*UTI 24 hr and model 24 hr groups*

**Figure 6:**
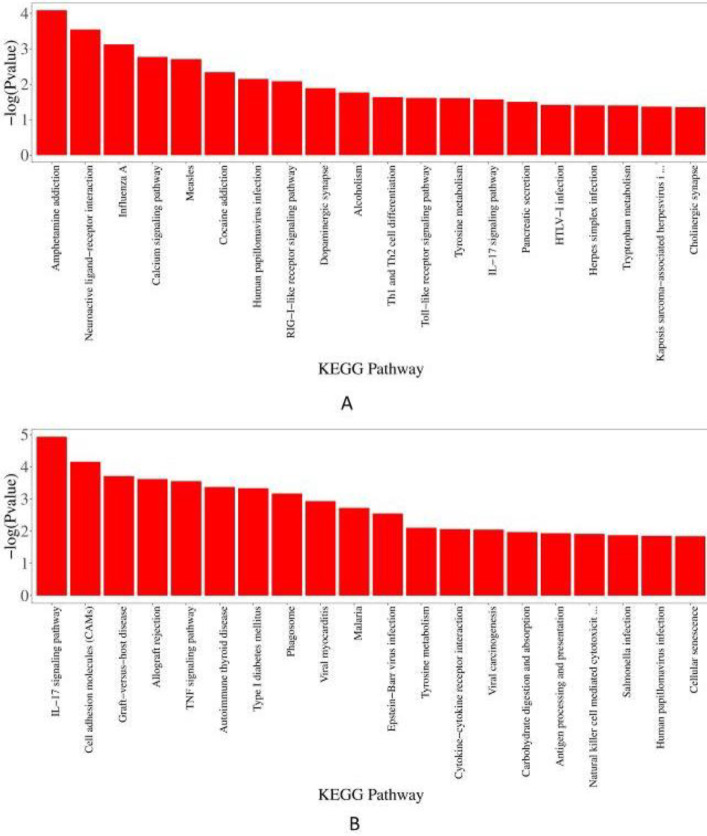
KEGG pathway analysis. (A) Bar plot of down-regulated DEGs between UTI 24h and model 24 hr groups; (B) Bar plot of up-regulated DEGs between UTI 24 hr and model 24 hr groups. KEGG: Kyoto Encyclopedia of Genes and Genomes; UTI: ulinastatin; DEGs: differential expression

For down-regulated DEGs (*P*<0.05, Figure 6A), the results showed that the infectious diseases and immune systems took up more of these enriched pathways. Moreover, there were also substance dependence pathways, nervous system pathways, neuroactive ligand-receptor interaction, etc. According to the up-regulated DEGs (*P*<0.05, Figure 6B), seven Immune systems (IL-17 signaling pathway, graft-versus-host disease, allograft rejection, etc) and several infectious diseases showed more important roles. Other several pathways included the signaling molecules and interaction pathways, TNF signaling pathway, etc. 

*UTI 72 hr and model 72 hr groups*

**Figure 7:**
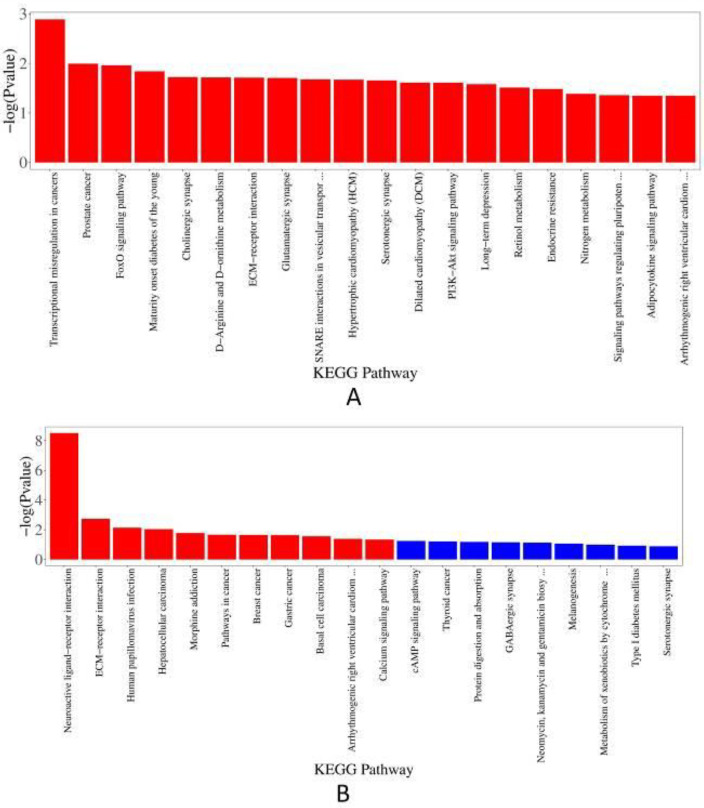
KEGG pathway analysis. (A) Bar plot of down-regulated DEGs between UTI 72 hr and model 72 hr groups; (B) Bar plot of up-regulated DEGs between UTI 72 hr and model 72 hr groups. KEGG: Kyoto Encyclopedia of Genes and Genomes; UTI: ulinastatin; DEGs: differential expression genes

Regarding the down-regulated DEGs (*P*<0.05, Figure 7A), there were two cancer pathways enriched, including transcriptional misregulation in cancers and prostate cancer. In addition, the results included some nervous system pathways, cardiovascular disease pathways, fluid shear stress, atherosclerosis, etc. And in the up-regulated DEGs (*P*<0.05, Figure 7B), there were only eleven enriched pathways significantly, which were enriched in two signaling molecules and interaction pathways (neuroactive ligand-receptor interaction, ECM-receptor interaction) and several cancer pathways. Others were related to human papillomavirus infection, morphine addiction, etc.

***UTI can regulate inflammation response of brain injury after CA/ROSC***

**Figure 8:**
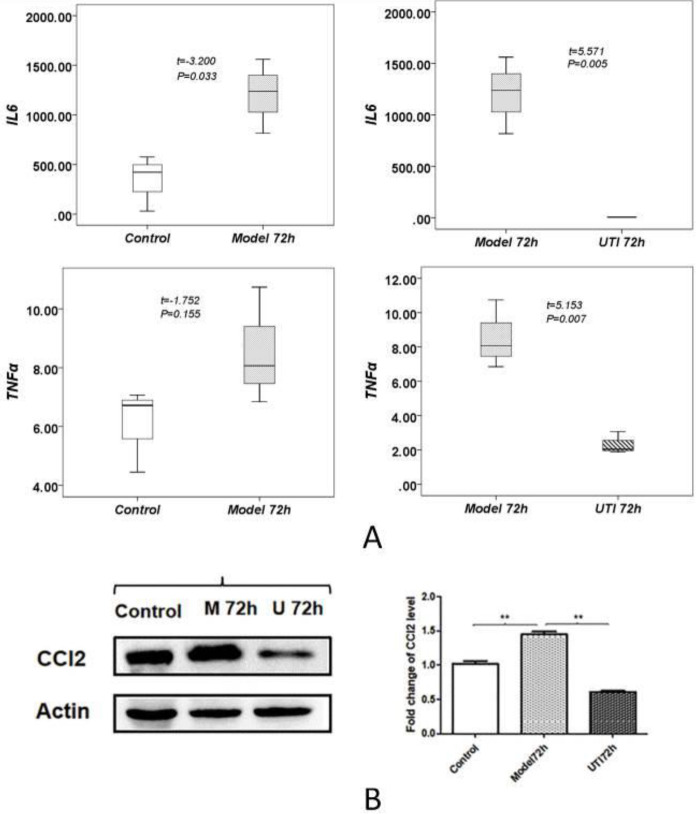
Verification. (A) Cytokine analysis of IL-6 and TNF-α between different groups; (B) WB of CCl2; **: *P*<0.05

WB: Western blot; UTI: ulinastatin; CCl2: C-C motif chemokine ligand 2

The results showed that the serum levels of IL-6 and TNF-α increased at 72 hr after modeling and decreased significantly after UTI intervention (*P*<0.05, Figure 8A). WB study results showed that the protein expression of CCl2 increased in the model 72 hr group while decreasing after UTI therapy (*P<*0.05**, **Figure 8B).

***UTI can improve the neuronal function of brain injury after CA/ROSC***

**Figure 9:**
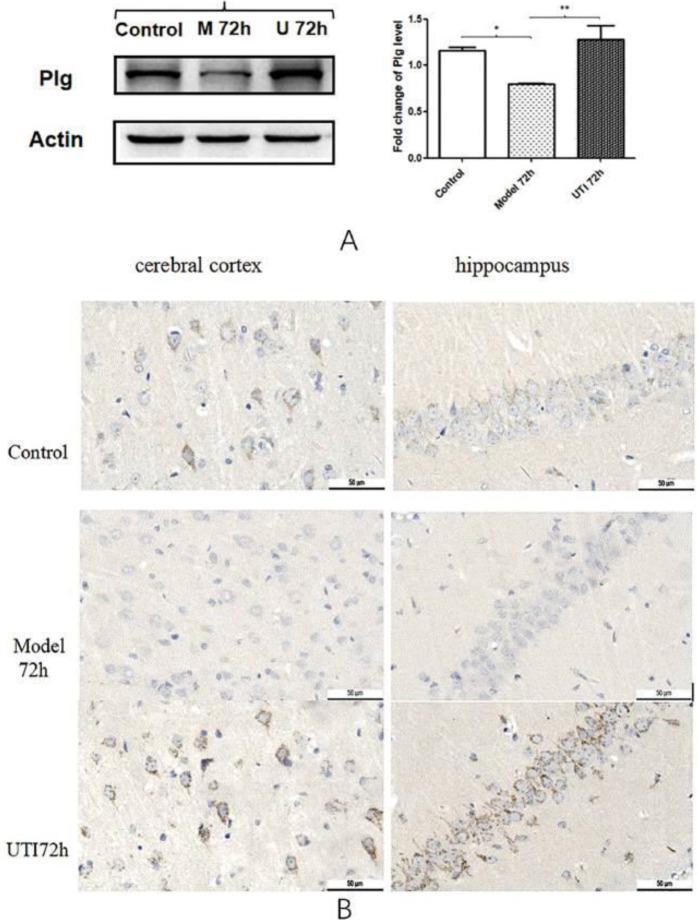
Verification. (A) WB of plg; (B) IHC of plg in cerebral cortex and hippocampus, 40X

WB: Western blot; IHC: immunohistochemistry; UTI: ulinastatin; Plg: plasminogen; *: *P*<0.05; **: *P*<0.01

The protein expression of plg showed a consistent trend both in the WB and IHC studies, which decreased at 72 hr after modeling and significantly increased after UTI intervention (*P*<0.05, Figure 9A). In addition, the IHC also found that the location of plg expression was mainly in the cytoplasm/membrane of neuronal cells, which was also consistent with the CC of GO enrichment analysis results (Figure 9B).

Group G1: model group; Group G2: UTI group; UTI: ulinastatin; DEGs: differential expression genes.

## Discussion

Brain injury after CA/ROSC has always attracted researchers’ attention because of its high mortality and disability rates, but there is no effective therapy at present (20). As mentioned in the introduction, UTI is a urinary protease inhibitor that has been increasingly recognized for its effectiveness for brain injury after CA/ROSC in recent years while its mechanisms are rarely studied. 

First of all, we can directly find that there were two important time nodes in the model and UTI groups by observing the survival rates of rats, which were 1~3 days and one week after modeling. Especially, the mortality of rats was relatively high at the time node of 1~3 days after modeling, which was also the main reason why we chose these two time points in the later studies. This is also consistent with a past study (21). And it is obvious that UTI can improve the survival rate of rats after CA/ROSC. Inflammation is a very important part of innate immunity and is regulated in many steps. Both IL-6 and TNF-α are critical anti-inflammatory factors in the inflammatory response, especially for TNF-α (22, 23). TNF-α was found to be implicated in numerous autoimmune diseases and central nervous system diseases (24, 25). IL-6 is also considered a cytokine with pleiotropic functions in both immune and nonimmune cells, which can influence many immune and physiological processes (26). Our results showed that the serum levels of IL-6 and TNF-α increased at 72 hr after modeling and decreased significantly after UTI intervention, which indicated the obvious anti-inflammatory effect of UTI. 

According to the results of RNA-seq, the enriched neuroactive ligand-receptor interaction pathway significantly increased after 72 hr of UTI treatment. Meanwhile, the IL-17 signaling pathway significantly increased at 24 hr and decreased at 72 hr after treatment with UTI. The neuroactive ligand-receptor interaction pathway, a collection of receptors located on the plasma membranes, is involved in the transduction of signals from the extracellular environment into cells. Neuroactive ligand influences neuronal function by binding to intracellular receptors that can act as transcription factors and regulate gene expression (27-29). Previous studies have pointed out that this pathway plays an important regulatory role in some neurological diseases, such as Autism (29) and Parkinson’s Disease (30). Regarding the IL-17 signaling pathway, we focused on the balance between the pathogenic and protective roles of IL-17 in autoimmune disease (31). IL-17 is the founding member of a novel family of inflammatory cytokines, its pro-inflammatory properties are key to the host-protective capacity. In addition, IL-17 is also vital for barrier protection and tissue regeneration. While transient and regulated IL-17 activity elicits physiological responses for host defense and tissue repair, chronic IL-17 activity orchestrates pathogenic responses that promote autoimmunity (32, 33). In general, UTI has shown efficacy in the treatment of brain injury after CA/ROSC, which is mainly by inducing autoimmune response, producing an anti-inflammatory effect, and regulating neuronal function. Importantly, to the best of our knowledge, no literature has identified the expression profiles of whole-brain mRNA in the UTI therapy for brain injury after CA/ROSC. 

Simultaneously, we respectively selected one significant DEG (CCl2 and plg) in the above two enriched pathways and verified them by WB and IHC techniques. CCl2 and its receptor CCR2 signaling mediate pathological post-ischemic inflammatory response by not only inducing leukocyte recruitment but also disrupting the blood-brain barrier and leukocyte adhesion to brain endothelial cells. Many studies have shown that CCl2-CCR2 signaling plays an important role in inducing neuroinflammation and promoting neuronal apoptosis (34-36). Results showed that the protein expression of CCl2 increased in the model 72 hr group and decreased significantly after UTI treatment in WB. As for plg, it is the precursor of the fibrinolytic enzyme. In normal organisms, the fibrinolytic enzyme exists in a prozyme state, and only through the action of fibrinolytic activator can the fibrinolytic enzyme become active. A new study suggests that plg may play a role in promoting angiogenesis in the peri-ischemic brain tissue, which contributes to functional recovery after ischemic stroke (37). Besides, previous studies have also shown that plg can inhibit neuronal apoptosis and axonal injury and promote white matter integrity and functional recovery after traumatic brain injury (38, 39). The protein expression of plg decreased in the model 72 hr group and increased significantly after UTI treatment in the WB. The above verification results were almost consistent with the GO and KEGG enrichment analysis of RNA-seq, which also indicates that UTI can be beneficial to produce protective effects for brain injury after CA/ROSC by improving neuronal function and modulating immune-inflammatory response. However, some other further studies regarding this are needed in the future.

## Conclusion

UTI therapy can improve the survival rate and neuronal function in the rat model of brain injury after CA/ROSC. And the main mechanisms are regulation of immune-inflammatory response and the signaling molecules and interaction, such as the IL-17 signaling pathway and neuroactive ligand-receptor interaction pathway.

## Authors’ Contributions

LXZ, HZ, and XJB Conceived the study or design; TTZ, WW, and YYH Processed and collected data, and performed experiments; MYY, XJB, and YL Analyzed and interpretated the results; XJB and MYY Provided draft manuscript preparation and visualization; JX and TM Critically revised or edited the article; HZ and LXZ Approved the final version to be published; HZ and LXZ Provided supervision and funding acquisition.
